# Supplementary material for: Chromothripsis during telomere crisis is independent of NHEJ, and consistent with a replicative origin
Source: Genome Res. 2019 May;29(5):737–49. doi: 10.1101/gr.240705.118 (PMC6499312; doi:10.1101/gr.240705.118)
Supplement: Supplemental Material [file supp_gr.240705.118_Supplemental_file_1.zip › contigs/annotated_contigs/DB56/contig.3.DB56_length_853_mean_cov_8.98710433763.docx]

**DB56_length_853_mean_cov_8.98710433763**

```
GGCGGGGTGG|GGGGGGGGGGGGGCGGGGGGGGGGGGGGGCCTGGGGGGGGGGGGGGGG|CCCCCGGGGCCCGGGGGGGG|GGGTGGGG
           >chr2:33141624-33141670 + E=6e-02 p=0e+00                           >chr14:
CTCGGGGGCAGGGCTGTGGCCTCGCTCACCCCTGTGCTGTGCCTTGCCTACAGGTGAAGTGGATCTTCTCCTCGGTGGTGGACCTGAAG
106203910-106204243 - E=2e-185
CAGACCATCATCCCCGACTACAGGAACATGATCGGACAGGGGGCCTAGGGCCACCCTCTGCGGGGTGTCCAGGGCCGCCCAGACCCCAC

ACACCAGCCATGGGCCATGCTCAGCCACCACCCAGGCCACACCTGCCCCCGACCTCACCGCCCTCAACCCCATGGCTCTCTGGCCTCGC

AGTTGCCCTCTGACCCTGACACACCTGACACGCCCCCCTTCCAGACCCTGTGCATAGC|T|AGAAGGAGCCTGGAAGGGCTAGAGAACA
                                                            >chr7:149197424-149197865 +
TGAGACCACATGTCAACTCAAGGTGTCAGGGAGGCAGGAAGTGTGTGGTTCATACTGCACGTAGACTGTGTAGATGTAGGGACTATGGA
 E=2e-250
CCCATGCACCCCTATGAGTGCTTCCTATACTCCAGCAGGTACAGCAGAACAGGTGGACCTCTATTTTTCCAGCTGCTCCCTTAGGCCTG

AGCTTTTCCCCTGGGACCAGGCACACAGCCTAAAAGTTCAGGCTTGGGGTTTTCAAAAAGACTGTGGTAATCATTGCTGCCCACTAAAT

ATTTCCAGCTGGTTTCTCTTGAAGACCATGGCTGGATGGCACTTCCTGGTTTCTTCTGGCTGGGTTGGACCATGTGACAAGTTCTGGCT

GATGTTTTTTCTTTCCCTCTACTATGGTAACCCAGCAATATTTGAGGAGGTGGGTGC
```
